# Atypical Presentation of Chronic Left Anterior Descending Occlusion: Heart Failure Without Angina as the Initial Manifestation

**DOI:** 10.7759/cureus.98903

**Published:** 2025-12-10

**Authors:** Wiktoria M Gembala, Julia K Marczuk, Iwona Kobielusz-Gembala

**Affiliations:** 1 Faculty of Medicine, Jagiellonian University Medical College, Krakow, POL; 2 Cardiology Department, Medicome Clinic Oswiecim, Oswiecim, POL

**Keywords:** chronic total occlusion, collateral circulation, coronary artery disease, heart failure, young patient

## Abstract

We present the case of a middle-aged patient with few conventional cardiovascular risk factors (no hypertension, diabetes, or smoking), whose first manifestation of chronic left anterior descending (LAD) artery occlusion was severe heart failure accompanied by pulmonary edema. The patient was admitted with progressively worsening dyspnea at rest for about two weeks and edema of the lower limbs. Echocardiography revealed a severely reduced left ventricular ejection fraction (~18%), along with fluid in both pleural cavities and signs of pulmonary congestion. Cardiac markers (troponin, CK-MB) did not confirm an acute infarction. After clinical stabilization, coronary angiography was performed, revealing a proximal LAD chronic total occlusion (CTO) with classic angiographic features, including a blunt proximal stump, absent antegrade flow, and well-developed collateral filling of the distal LAD from the dominant right coronary artery. The patient was scheduled for elective revascularization of the LAD CTO, which had not yet been performed. The reversed order of symptoms (heart failure without angina or acute infarction) and the relative paucity of traditional risk factors in this middle-aged patient represent an unusual clinical presentation. This case highlights the importance of considering an ischemic etiology of heart failure even when symptoms are atypical or non-anginal.

## Introduction

Chronic total occlusion (CTO) of a coronary artery is detected in 15-30% of patients undergoing angiography, especially in populations with extensive atherosclerotic changes [1]. Most CTOs involve the right coronary artery (RCA), whereas CTO of the left anterior descending (LAD) artery is less common but clinically more consequential due to the large myocardial territory it supplies. CTOs typically occur in older patients with multiple cardiovascular risk factors (e.g., hypertension, dyslipidemia, diabetes, tobacco use) [2]. Such patients typically report classic coronary symptoms, such as angina pectoris or symptoms of acute coronary syndrome [2]. Meanwhile, patients with diagnosed heart failure often exhibit symptoms of chronic coronary disease or ischemic cardiomyopathy, but it is rare for heart failure to manifest first as the initial sign of coronary disease [2]. This case report has educational value, as it presents an atypical sequence of symptoms - severe heart failure as the first manifestation of chronic LAD occlusion - in a middle-aged patient with few traditional risk factors and no prior ischemic events and without angina or evidence of acute infarction.

## Case presentation

The patient reported no prior history of exercise intolerance, exertional dyspnea, chest discomfort, or cardiovascular symptoms. No orthopnea or paroxysmal nocturnal dyspnea was documented. His functional capacity had been normal until approximately two weeks before admission, when rapidly progressive dyspnea and lower-limb edema developed, consistent with an acute decompensated presentation rather than chronic symptomatic decline. A 40-year-old man with no known chronic diseases presented with progressively worsening dyspnea over about two weeks, initially on exertion and later at rest. He also complained of lower limb edema. He denied chest pain, palpitations, or loss of consciousness. His medical history was negative for arterial hypertension, diabetes, smoking, or myocardial infarction, and he reported no family history of premature coronary artery disease. On physical examination, a cardiac murmur, crackles at the lung bases, and generalized edema were noted. On admission, his blood pressure was 127/103 mmHg and heart rate was 92 beats/min. A cardiac murmur was noted; however, its characteristics (systolic vs. diastolic, grade, location, radiation) were not documented in the available records. No documentation was available regarding the presence of S3 or S4 gallops, jugular venous distension, or hepatomegaly.

Laboratory tests showed a markedly elevated NT-proBNP (2,267 pg/mL), confirming severe decompensated heart failure. Cardiac markers (CK-MB, troponin) were within normal limits, suggesting the absence of acute myocardial injury (no infarction). A single high-sensitivity troponin measurement was available (0.023 µg/L, within the reference range); serial troponin testing was not documented. The lipid profile revealed markedly low HDL cholesterol (19 mg/dL), which represents an atherogenic abnormality despite otherwise moderate LDL levels (66 mg/dL) and low total cholesterol (112 mg/dL). Liver enzymes were mildly abnormal, with an elevated alanine aminotransferase (ALT 95 U/L, later decreasing to 66 U/L) and normal aspartate aminotransferase (AST 38 U/L). Thyroid function testing was not documented in the available records. Other tests, including glucose and creatinine levels, were within normal range.

**Figure 1:**
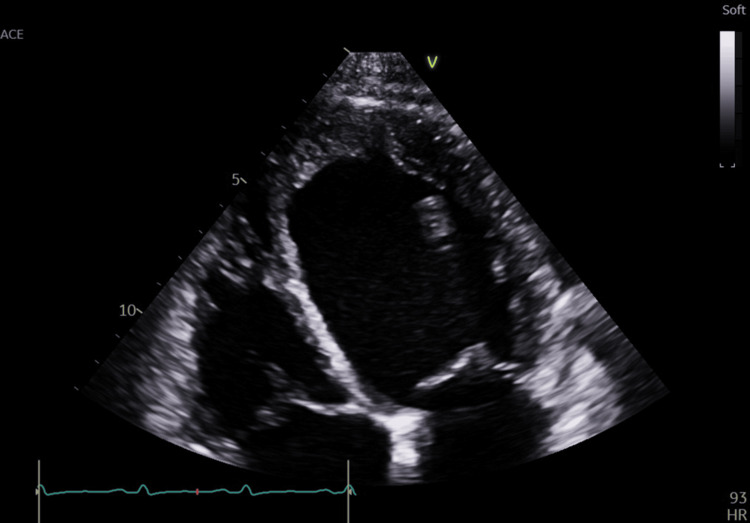
Apical four-chamber view showing severe left ventricular systolic dysfunction Apical four-chamber transthoracic echocardiographic view obtained in systole demonstrating severe global left ventricular systolic dysfunction, with markedly reduced cavity obliteration and minimal inward myocardial motion. The left ventricle and left atrium are significantly dilated. The interventricular septum appears thinned, akinetic, and hyperechogenic, consistent with chronic ischemic injury. The right-sided chambers are also enlarged. Overall, the image illustrates advanced dilated remodeling with profoundly impaired contractility, corresponding to the patient’s severe heart failure presentation.

**Figure 2:**
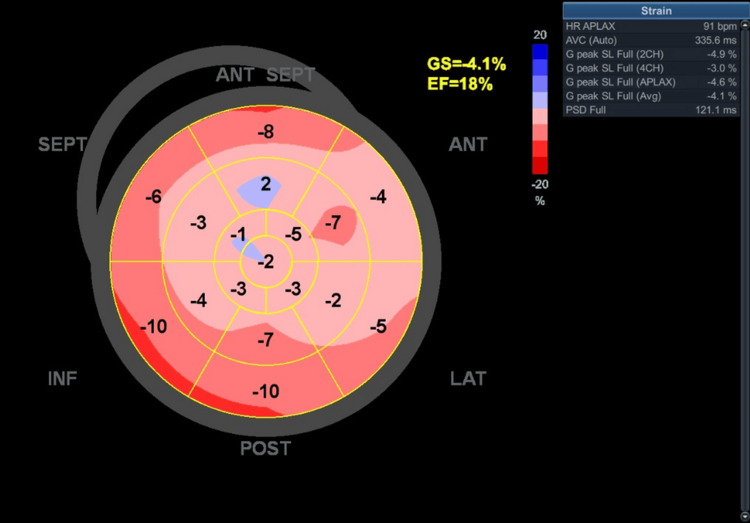
Bull’s-eye plot demonstrating severe reduction in global longitudinal strain Bull’s-eye plot of left ventricular global longitudinal strain showing severely reduced myocardial deformation, with a global strain value of –4.1%. Most myocardial segments exhibit markedly impaired or near-absent strain, with the most pronounced abnormalities localized in the anterior and septal regions. The uniform red-to-pink pattern reflects diffuse systolic dysfunction, consistent with the severely reduced left ventricular ejection fraction (18%). This strain map supports the presence of advanced chronic ischemic cardiomyopathy. ANT, anterior; SEPT, septal; LAT, lateral; INF, inferior; POST, posterior

**Figure 3:**
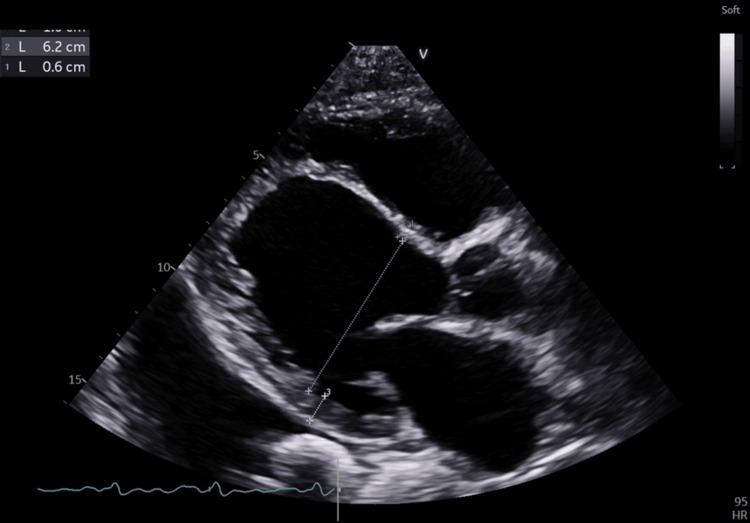
Parasternal long-axis echocardiographic view showing severe left ventricular dilatation and global systolic dysfunction Parasternal long-axis transthoracic echocardiographic view demonstrating a markedly dilated left ventricle with severely reduced systolic thickening and minimal inward myocardial motion. The interventricular septum appears thinned and akinetic, consistent with chronic ischemic remodeling, while the posterior wall exhibits only limited contractility. The left atrium is enlarged, and the aortic root and mitral valve structures remain anatomically preserved, although functional impairment is present secondary to ventricular dilatation. These findings illustrate advanced dilated cardiomyopathy with profound systolic dysfunction, correlating with the patient’s severely reduced ejection fraction.

An electrocardiogram (ECG) was performed, demonstrating pathological Q waves in leads I and aVL, consistent with high-lateral (anterolateral) involvement, a pattern often associated with the diagonal branch of the LAD or the obtuse marginal branch of the left circumflex artery (LCx). Low-voltage limb leads were also present, a nonspecific finding commonly seen in dilated cardiomyopathy. Small R waves were present in all precordial leads (V1-V6), reflecting poor R-wave progression, a finding that may indicate prior anterior myocardial injury or chronic ischemia. No Q waves were present in the anterior precordial leads. Echocardiography revealed severely reduced global left ventricular systolic function (ejection fraction [EF] ~18%, assessed using the Simpson biplane method; global longitudinal strain [GLS] −4.1%, obtained via vendor-specific speckle-tracking analysis) with generalized hypokinesia (Figures 1, 2). Although the patient denied any prior history of myocardial infarction or anginal symptoms, the echocardiographic findings - including akinesis, thinning, and increased echogenicity of the anterior interventricular septum - suggest features consistent with chronic ischemic injury, possibly related to a silent or remote myocardial infarction. Both ventricles and atria were markedly enlarged (Figure 3). Pleural effusions were identified on ultrasound; however, thoracentesis was not performed, and therefore fluid characterization (transudative vs. exudative) was not available. No chest X-ray or POCUS (point-of-care ultrasound) images were obtained for documentation. Cardiac magnetic resonance imaging (MRI) had not yet been performed at the time of evaluation, and therefore tissue characterization studies were not available.

The working diagnosis was decompensated heart failure with reduced ejection fraction (HFrEF), likely of ischemic origin based on ECG and echocardiographic abnormalities, with no evidence of acute infarction. The patient’s symptoms corresponded to New York Heart Association (NYHA) class III/IV prior to treatment. Coronary angiography performed after clinical stabilization subsequently confirmed a proximal CTO of the LAD with well-developed collateral circulation. Guideline-directed medical therapy for HFrEF was initiated, including sacubitril/valsartan (ARNI), a beta-blocker, a mineralocorticoid receptor antagonist, and an SGLT2 (sodium-glucose co-transporter 2) inhibitor, along with loop diuretics and ivabradine. After stabilization of the clinical condition (intensive therapy with diuretics and vasodilators, weight loss of 8 kg, resolution of edema and pleural effusions), coronary angiography was performed. The radial artery approach revealed CTO of the proximal LAD and a well-developed collateral system (a prominent right ventricular and left ventricular branch of the right coronary artery supplying the apex and distal LAD) (Video 1). The circumflex branch of the left coronary artery (LCA) was narrowed distally (~80%). The lesion was located in a small-caliber distal segment (<1.5 mm), and no physiological assessment (FFR/iFR) was performed. Given the vessel size and anatomy, the LCx stenosis was managed conservatively without percutaneous coronary intervention (PCI). The right coronary artery was dominant with numerous branches. Given that the patient had isolated single-vessel disease with a proximal LAD CTO and otherwise preserved coronary anatomy, PCI was selected as the preferred revascularization strategy. Coronary artery bypass grafting was not pursued, as the remaining vessels were patent, the LCx lesion was distal and small-caliber, and there were no surgical indications. The conclusion of the study was to consider elective recanalization of the LAD CTO. At discharge, the patient was hemodynamically stable (blood pressure 110/72 mmHg, no edema) and was started on a full guideline-directed medical therapy regimen for HFrEF. This included sacubitril/valsartan 24/26 mg twice daily, bisoprolol 2.5 mg once daily, spironolactone 25 mg once daily, empagliflozin 10 mg once daily, loop diuretic therapy (torasemide 10 mg once daily), and ivabradine 5 mg twice daily. Secondary prevention therapy consisted of aspirin 75 mg daily and rosuvastatin 40 mg daily. Potassium supplementation (Kaldyum) was prescribed due to ongoing diuretic use. He was discharged with instructions to continue outpatient cardiology follow-up and to undergo planned CTO revascularization.

**Video 1:** Coronary angiography demonstrating proximal LAD chronic total occlusion with collateral RCA supply Coronary angiography showing a proximal chronic total occlusion of the LAD with a blunt proximal stump and absent antegrade flow. The distal LAD is visualized only through well-developed collateral vessels originating from the dominant RCA. A significant mid-segment stenosis of the circumflex artery is also observed, while the RCA remains widely patent. LAD, left anterior descending artery; RCA, right coronary artery

## Discussion

The presented case demonstrates an uncommon sequence of symptoms in coronary artery disease. According to major guidelines, patients with chronic coronary syndromes typically present with angina, exertional dyspnea, or evidence of myocardial ischemia, whereas heart failure usually develops later as a consequence of progressive ischemic injury or prior infarction [3] . Moreover, the European Society of Cardiology heart failure guidelines emphasize that heart failure as an initial manifestation is more characteristic of non-ischemic cardiomyopathies than of chronic coronary disease [4]. In our patient, the difference was that severe heart failure was the first manifestation of LAD CTO despite the absence of evidence for acute infarction based on negative cardiac biomarkers. However, the ECG and echocardiographic findings were consistent with possible prior silent myocardial injury.

Additionally, the described patient had relatively few traditional cardiovascular risk factors (no hypertension, diabetes, or smoking, and only dyslipidemia with markedly low HDL). Statistics indicate that CTO most frequently occurs in older individuals with hypertension, diabetes, or significant coronary atherosclerosis [2]. In the experience of the Cardiovascular Biobank study, patients with CTO were usually older, burdened with risk factors, and had worse left ventricular function [2]. Our patient was 40 years old, a non-smoker, with a normal BMI after treatment and a markedly low HDL cholesterol level - an atherogenic abnormality - but otherwise few traditional risk factors, which is uncommon in the context of CTO. It should be noted that the patient presented with marked fluid overload on admission, including peripheral edema and bilateral pleural effusions, and had an 8-kg weight reduction during hospitalization; therefore, the admission BMI was artificially elevated, whereas the post-diuresis BMI more accurately reflected his true baseline status.

Also unique was the presence of a strong collateral circulation. The development of a rich collateral network in the setting of CTO can limit the extent of myocardial necrosis. As described by Bryniarski et al., well-developed collateral vessels may help preserve viability within the region supplied by the occluded artery [5]. However, in this patient, the protection was only partial, as evidenced by septal thinning, akinesis, and increased echogenicity on echocardiography. This likely limited the extent of transmural necrosis and preserved viability in some myocardial segments despite the presence of severe systolic dysfunction. The presence of well-developed collateral circulation may also explain the absence of typical angina, as chronic ischaemia can be partially compensated by alternative perfusion pathways.

It should be emphasized that chronic CTO is associated with an increased risk of complications and a worse prognosis in heart failure. In the study by Behnes et al., CTO was found in 17% of patients hospitalized for heart failure (EF 41-49%), and it was associated with significantly worse treatment outcomes than in patients without CTO [6]. Our case supports consideration of revascularization in patients with CTO, particularly when it coexists with severe left ventricular dysfunction, while recognizing that current evidence for prognostic benefit remains limited and mixed. Formal ischemia or viability testing had not yet been performed at the time of evaluation; however, further assessment (e.g., cardiac MRI) was planned. Therefore, the recommendation for CTO revascularization was based on anatomical findings and guideline-extrapolated considerations rather than definitive viability imaging. In the treatment protocol, the patient was scheduled for elective angioplasty of the LAD CTO, which aligns with recommendations for revascularization to improve flow and myocardial function.

Comparing with the literature, sporadic cases of “silent” coronary disease ending with sudden acute heart failure have been described. In the aforementioned study, 33% of patients with heart failure and CTO died within 30 months, whereas it was 19% for patients without CTO [6]. Although our patient was relatively young and with no evidence of recent infarction, this underscores the seriousness of the diagnosis and the rationale for considering interventional therapy. Additionally, the presence of pathological Q waves in leads I and aVL is consistent with high-lateral involvement, a pattern often associated with disease of the diagonal branch of the LAD or the obtuse marginal branch of the LCx. Such Q waves more commonly reflect prior myocardial infarction rather than mere ischemia, supporting the possibility of remote myocardial injury in this patient.

It is also worth noting that recently published case reports emphasize that chronic occlusions of coronary arteries can manifest atypically - even with severe left ventricular dysfunction and without chest pain - which further underscores the importance of diagnosing CTO in patients with unexplained HFrEF [7]. For example, Habib et al. described the case of a patient with CTO of the left main coronary artery who exhibited no prior anginal symptoms and presented with sudden cardiac arrest as the initial manifestation of coronary disease, illustrating that CTO may remain clinically silent due to collateral perfusion until abrupt decompensation occurs [8]. Although the vascular territory, age, and clinical presentation in that report differ from our case, it supports the possibility that CTO may initially present with heart failure rather than angina, as observed in our patient.

## Conclusions

This case demonstrates an atypical presentation of coronary artery disease; in a middle-aged patient without anginal symptoms and without evidence of acute infarction, severe heart failure with pulmonary edema was the first manifestation of LAD CTO. Although the presence of well-developed collateral vessels may have limited the extent of ischemic injury, the echocardiographic findings of septal akinesis, thinning, and increased echogenicity indicate that myocardial protection was only partial and that chronic scarring was already present. From a clinical perspective, several points emerge. Firstly, Even in middle-aged patients with acute heart failure, an ischemic cause should be considered, especially when echocardiography reveals evidence of segmental ischemia. Secondly, in severe HFrEF without an obvious cause, consideration should be given to coronary angiography, as identifying and, where appropriate, revascularizing significant coronary stenoses may improve symptoms and possibly prognosis in selected patients. Thirdly, in patients with CTO, risk-stratification studies (such as exercise testing or perfusion imaging) may be considered to assess myocardial viability and determine which territories could benefit from revascularization; however, such testing was not performed in this case.
